# Supplementary material for: Pre-/-post-analyses of a feasibility study of a peer-based club intervention among people living with type 2 diabetes in Vietnam’s rural communities
Source: PLoS One. 2023 Nov 28;18(11):e0290355. doi: 10.1371/journal.pone.0290355 (PMC10684024; doi:10.1371/journal.pone.0290355)
Supplement: S1 Table — (DOCX) [file pone.0290355.s001.docx]

## Supporting information 1. Eleven leaflets using as educational materials in diabetes classes and clubs

| *Leaflet 1* | *Diabetes – things to know* |
| --- | --- |
| *Leaflet 2* | *Diabetes treatment* |
| *Leaflet 3* | *Balanced nutrition for diabetics* |
| *Leaflet 4* | *Meal plan* |
| *Leaflet 5* | *Diabetes management* |
| *Leaflet 6* | *Initial treatment for hyperglycemia/hypoglycemia* |
| *Leaflet 7* | *Diabetes foot problems* |
| *Leaflet 8* | *Foot care for diabetes* |
| *Leaflet 9* | *Physical activities for diabetics* |
| *Leaflet 10* | *Diabetes control on some special occasions* |
| *Leaflet 11* | *Living healthy in Tet’s holiday* |
