# Supplementary material for: Pre-/-post-analyses of a feasibility study of a peer-based club intervention among people living with type 2 diabetes in Vietnam’s rural communities
Source: PLoS One. 2023 Nov 28;18(11):e0290355. doi: 10.1371/journal.pone.0290355 (PMC10684024; doi:10.1371/journal.pone.0290355)
Supplement: S2 File — (DOCX) [file pone.0290355.s004.docx]

## Supporting information 2. The questionnaire used in the paper

| STUDY INFORMANT ID No:_______  **Intervention for people living with diabetes**  **QUESTIONNAIRE**  NAME: _______________________  VILLAGE: _______________________  COMMUNE: ___________________________  DISTRICT: VŨ THƯ – PROVINCE: THÁI BÌNH  IDENTIFICATION NUMBER (IF KNOWN): ___________________  HEALTH INSURANCE NUMBER: ___________________  DATE OF INTERVIEW (dd/mm/yyyy): …..…/……../2021   \| 000. RECORD THE TIME THE INTERVIEW BEGINS \| Hour [ ][ ] (24 h)  Minutes [ ][ ] \| \| --- \| --- \| \| 1. Name of interviewer \|  \| \| 1. Name of supervisor \|  \| |
| --- | --- | --- | --- | --- | --- | --- |

Thank you so much for taking time to talk with us. As I have already told you, we are conducting a research project about how it is to live with diabetes in Vietnam. As you may know, diabetes is a chronic life-long disease that often requires daily care. Researches from other countries have shown that 95% of all tasks related to diabetes management take part outside the health system. Therefore, it is quite common that having diabetes is not only a matter for the patient, but also for the patient’s family members and friends. Research has shown that family members as well as community especially diabetics community are very important for the management of diabetes. Diabetes is a new disease in Vietnam and we think that diabetes creates a huge demand on the patients, families, communities and health systems but we actually do not have any research about it. Therefore, we would like to ask you about your experience of living with diabetes, how much the disease requires from you and how you feel about, the different kinds of support that you need more to improve your health, quality of life and help you control diabestes better. We hope this research can help us with knowledge of how the health system and community can help patients living with diabetes carry the burden of diabetes.

| SECTION 1THE RESPONDENT INFORMATION | | | |
| --- | --- | --- | --- |
| QUESTIONS & FILTERS | | CODING CATEGORIES | NOTES |
| If you don’t mind, I would like to start by asking you a little about yourself and the household that you live in.  Interviewer circles the selected option(s). | | |  |
| 101 | Gender of respondent | \| MALE \| FEMALE \| \| --- \| --- \| \| 0 \| 1 \| |  |
| 102 | Date and year of birth?  ‘Unknown’ date is marked as 99  ‘Unknown’ month is marked as 99  ‘Unknown’ year is marked as 9998  ‘Refused/no answer’ is marked as 9999 | DATE OF BIRTH: ___/_____/_____ (DD/MM/YYYY)  LUNAR DATE OF BIRTH: ___/_____/_____ (DD/MM/YYYY) |  |
| 102a: | If the date of the birth unknown:  How old are you? | \| AGE (YEARS) \| DON’T KNOW \| REFUSED/NO ANSWER \| \| --- \| --- \| --- \| \|  \| 888 \| 999 \| |  |
| 103 | What is the highest level of education that you finished?  **MARK HIGHEST LEVEL**. | \| NEVER ATTENDED SCHOOL \| 1 \| \| --- \| --- \| \| PRIMARY SCHOOL \| 2 \| \| SECONDARY SCHOOL \| 3 \| \| HIGH SCHOOL \| 4 \| \| UNIVERSITY/COLLEGE \| 5 \| \| POSTGRADUATE \| 6 \| \| DON’T KNOW/DON’T REMEMBER \| 7 \| \| REFUSED/NO ANSWER \| 8 \| |  |
| 104 | What is your **MAIN** current occupation? | \| UNEMPLOYED \| 1 \| \| --- \| --- \| \| STAY-AT-HOME WIFE/HUSBAND \| 2 \| \| FARMER \| 3 \| \| SMALL TRADE (SELF-BUSINESS) \| 4 \| \| WORKER \| 5 \| \| GOVERNMENT EMPLOYEE \| 6 \| \| PRIVATE COMPANY/ORGANISATION EMPLOYEE \| 7 \| \| RETIRED \| 8 \| \| STUDENT \| 9 \| \| OTHER \| 10 \| \| REFUSED/NO ANSWER \| 11 \|   104_1a: OTHER (SPECIFY)____________________________ |  |
| 105 | How would you yourself assess the economic situation of your household?  (Read up options) | \| POOR \| 1 \| \| --- \| --- \| \| NEAR POOR \| 2 \| \| MEDIUM \| 3 \| \| WEALTHY \| 4 \| \| DON'T KNOW \| 5 \| \| REFUSED/NO ANSWER \| 6 \| | **POOR , NEAR POOR**  **⇒ HAVE BOOK** |
| 106 | Do you regularly attend a group, organization or association? | \| NO \| YES \| REFUSED/ NO ANSWER \| \| --- \| --- \| --- \| \| 0 \| 1 \| 9 \| |  |
| 106a | IF YES:  What kind of group, organization or association?  **(MULTIPLE ANSWERS ALLOWED)**  IF NO, PROMPT:  Organizations like women’s or community groups, religious groups or political associations. | \| 106a_1 \| WOMEN’S UNION \| 1 \| \| --- \| --- \| --- \| \| 106a_2 \| FARMERS’ UNION \| 2 \| \| 106a_3 \| YOUTH UNION \| 3 \| \| 106a_4 \| AGING UNION \| 4 \| \| 106a_5 \| RELIGIOUS ORGANIZATION \| 5 \| \| 106a_6 \| VETERANS’ ORGANIZATION \| 6 \| \| 106a_7 \| COMMUNIST PARTY \| 7 \| \| 106a_8 \| CULTURAL ASSOCIATION \| 8 \| \| 106a_9 \| HEALTH CLUB (e.g. yoga, meditation) \| 9 \| \| 106a_10 \| DIOXIN VICTIM ASSOCIATION \| 10 \| \| 106a_11 \| RETIREMENT ASSOCIATION \| 11 \| \| 106a_12 \| DIABETES CLUB \| 12 \| \| 106a_13 \| OTHER \| 13 \| \| 106a_14 \| REFUSED/NO ANSWER \| 14 \|   106a_13a: IF OTHER, SPECIFY:____________________________ |  |

| **SECTION 2**  **SOCIAL NETWORK** | | | |
| --- | --- | --- | --- |
| I would like to ask some questions about your family | | | |
| 201 | Are you married? If yes, do you live together?  **(ONLY ONE ANSWER ALLOWED)** | \| SINGLE \| 1 \| \| --- \| --- \| \| CURRENTLY MARRIED AND LIVING TOGETHER \| 2 \| \| CURRENTLY MARRIED BUT LIVING APART \| 3 \| \| LIVING TOGETHER, BUT NOT MARRIED \| 4 \| \| DIVORCED / SEPARATED \| 5 \| \| WIDOWED \| 6 \| \| REFUSED/NO ANSWER \| 7 \| |  |
| 202 | Do you have children? (only living children) | \| NO \| YES \| Number of sons \| Number of daughters \| REFUSED/NO ANSWER \| \| --- \| --- \| --- \| --- \| --- \| \| 0 \| 1 \|  \|  \| 9 \| | IF NO ⇒ 203 |
| 203 | What is the total number of persons living in your household?  **(EXCLUDING YOU)**  (including domestic servants if they sleep 5 nights a week or more in the household and visitors if they have slept 5 nights or more a week in the household for the past 4 weeks) | \| NUMBER \| REFUSED/NO ANSWER \| \| --- \| --- \| \|  \| 9 \| | **IF LIVES ALONE** ⇒ 204 |
| 203a | Who among these lives in your household?  If “Yes”, put the number in the column under “YES”.  (Read up all options)  **(MULTIPLE ANSWERS ALLOWED)** | \|  \|  \| YES  (INSERT NUMBER) \| \| --- \| --- \| --- \| \| 203a_1 \| SPOUSE \|  \| \| 203a_2 \| MOTHER \|  \| \| 203a_3 \| FATHER \|  \| \| 203a_4 \| MOTHER-IN-LAW \|  \| \| 203a_5 \| FATHER-IN-LAW \|  \| \| 203a_6 \| GRANDCHILDREN \|  \| \| 203a_7 \| SISTER \|  \| \| 203a_8 \| BROTHER \|  \| \| 203a_9 \| SISTER-IN-LAW \|  \| \| 203a_10 \| BROTHER-IN-LAW \|  \| \| 203a_11 \| DAUGHTER(S) \|  \| \| 203a_12 \| SON(S) \|  \| \| 203a_13 \| DAUGHTER(S)-IN-LAW \|  \| \| 203a_14 \| SON-IN-LAW \|  \| \| 203a_15 \| GRANDPARENTS/ GRANDPARENTS-IN-LAW \|  \| \| 203a_16 \| GREAT-GRANDCHILDREN \|  \| \| 203a_17 \| OTHER \|  \| \| 203a_18 \| REFUSED/NO ANSWER \|  \|   203a_17a: IF OTHER, SPECIFY:____________________________ |  |
| 204 | Do you have any children who live **outside** your household? | \| NO \| YES \| REFUSED/NO ANSWER \| \| --- \| --- \| --- \| \| 0 \| 1 \| 9 \| | **IF NO**  ⇒ 205 |
| 204a | If yes (children who live outside your household), where do they live?  **(MULTIPLE ANSWERS ALLOWED)** | \| 204a_1 \| THIS COMMUNE \| 1 \| \| --- \| --- \| --- \| \| 204a_2 \| ANOTHER COMMUNE \| 2 \| \| 204a_3 \| ANOTHER DISTRICT \| 3 \| \| 204a_4 \| ANOTHER PROVINCE OR CITY \| 4 \| \| 204a_5 \| DON’T KNOW/DON’T REMEMBER \| 5 \| \| 204a_6 \| REFUSED/NO ANSWER \| 6 \| |  |
| 205 | Do you know other people in your social network who have diabetes? | \| NO \| YES \| REFUSED/NO ANSWER \| \| --- \| --- \| --- \| \| 0 \| 1 \| 9 \| | **IF NO** ⇒ SECTION 3 |
| 205a | Who?  **(MULTIPLE ANSWERS ALLOWED)** | \| 205a_1 \| RELATIVES WITH WHOM I SHARE A HOUSEHOLD \| 1 \| \| --- \| --- \| --- \| \| 205a_2 \| RELATIVES OUTSIDE MY HOUSEHOLD \| 2 \| \| 205a_3 \| FRIEND/NEIGHBOR \| 3 \| \| 205a_4 \| OTHER \| 4 \| \| 205a_4 \| REFUSED/NO ANSWER \| 5 \|   205a_4a: IF OTHER, SPECIFY: __________________________ |  |

| **SECTION 4**  **MEDICATION AND USE OF HEALTH SERVICES** | | | | | | | | | |
| --- | --- | --- | --- | --- | --- | --- | --- | --- | --- |
| I would now like to ask a few questions about your medication and use of health services before and now. | | | | | | | | | |
| 401 | | Do you have health insurance? | | | \| NO \| YES \| REFUSED/NO ANSWER \| \| --- \| --- \| --- \| \| 0 \| 1 \| 9 \| | | | IF NO  **⇒ 403** | |
| 402 | | How many percentages of health insurance benefits do you get when you are check-ups as the regulation? (What level of the costs are covered by the health insurance?)  **(ONLY ONE ANSWER ALLOWED)** | | | \| 100% OF HEALTHCARE EXPENDITURE \| 1 \| \| --- \| --- \| \| 95% OF HEALTHCARE EXPENDITURE \| 2 \| \| 80% OF HEALTHCARE EXPENDITURE \| 3 \| \| OTHER \| 4 \| \| DON’T KNOW \| 8 \| \| REFUSED/NO ANSWER \| 9 \|   402_1a IF OTHER, SPECIFY:_____________________ | | |  | |
| 403 | | **When** did you get the diagnosis of diabetes?  Please year:  ‘‘Refused/no answer’ is marked as 99 | | | __________ | | ACCORDING TO THE PATIENT’S BOOK   - YES - NO |  | |
| 403a | | **If unknown year:** | | | \| > 20 YEARS \| 1 \| \| --- \| --- \| \| >10 – 20 YEARS \| 2 \| \| >5 – 10 YEARS \| 3 \| \| >1 - 5 YEARS \| 4 \| \| < 1 YEAR \| 5 \| \| DONT KNOW \| 6 \| \| REFUSED/NO ANSWER \| 7 \| | | |  | |
| 404 | | **Where** did you get the diagnosis of diabetes?  **(ONLY ONE ANSWER ALLOWED)** | | | \| NATIONAL-LEVEL HOSPITAL \| 1 \| \| --- \| --- \| \| PROVINCIAL HOSPITAL \| 2 \| \| TBUMP HOSPITAL \| 3 \| \| DISTRICT HOSPITAL \| 4 \| \| PRIVATE HOSPITAL/CLINIC \| 5 \| \| COMMUNE HEALTH STATION \| 6 \| \| OTHER (SPECIFY) \| 7 \| \| REFUSED/NO ANSWER \| 8 \|   404_1a: IF OTHER, SPECIFY: ______________________ | | |  | |
| 404a | | In last 6 months, where did you usually go for check-up?  (**ONLY ONE ANSWER ALLOWED)** | | | \| GENERAL HOSPITAL OF THAI BINH PROVINCE \| 1 \| \| --- \| --- \| \| GENERAL HOSPITAL OF THAI BINH CITY \| 2 \| \| TBUMP HOSPITAL \| 3 \| \| LAM HOA GENERAL HOSPITAL \| 4 \| \| HOANG AN GENERAL HOSPITAL \| 5 \| \| VU THU GENERAL HOSPITAL \| 6 \| \| OTHER \| 7 \| \| REFUSED/NO ANSWER \| 8 \|   404a_1a: IF OTHER, SPECIFY: ______________________ | | |  | |
| 405 | | **How often** do you usually go for check-up and treatment of diabetes?  **(ONLY ONE ANSWER ALLOWED)** | | | \| SEVERAL TIMES PER MONTH \| 1 \| \| --- \| --- \| \| EVERY MONTH \| 2 \| \| EVERY 3 MONTHS \| 3 \| \| EVERY 6 MONTHS \| 4 \| \| IRREGULARLY \| 5 \| \| OTHER \| 6 \| \| DON'T KNOW \| 7 \| \| REFUSED/NO ANSWER \| 8 \|   405_1a: IF OTHER, SPECIFY:______________________ | | |  | |
| 406 | | Do you have information about their last checkup + blood glucose measure in the booklet/paper from hospital? | | | \| NO \| YES \| \| --- \| --- \| \| 0 \| 1 \| | | | IF NO  ⇒ 407 | |
| 406a | | IF YES, please let me know the value at last checkup | | | \| ­­­­­­­­_____________________(mmol/L) \| \| --- \| | | |  | |
| 406b | | When did you get this value | | | ___/_____/_____ (DD/MM/YYYY) | | |  | |
| 407 | | Do you have information on HBA1C measure in the booklet/paper from hospital? | | | \| NO \| YES \| \| --- \| --- \| \| 0 \| 1 \| | | | IF NO  ⇒ 408 | |
| 407a | | IF YES, please let me know the value at last checkup | | | ______________________(%) | | |  | |
| 407b | | When did you get this value | | | ___/_____/_____ (DD/MM/YYYY) | | |  | |
| 408 | | Have you ever been **diagnosed with** the following medical conditions (other than diabetes)?  **(MULTIPLE ANSWERS ALLOWED)** | | | \| 408_1 \| HYPERTENSION \| 1 \| \| --- \| --- \| --- \| \| 408_2 \| EPILEPSY \| 2 \| \| 408_3 \| DEPRESSION \| 3 \| \| 408_4 \| TUBERCULOSIS \| 4 \| \| 408_5 \| LIVER AND KIDNEY DISEASE \| 5 \| \| 408_6 \| BONE AND JOINT PROBLEM \| 6 \| \| 408_7 \| CARDIO-VASCULAR DISEASE \| 7 \| \| 408_8 \| STROKE \| 8 \| \| 408_9 \| ANY OTHER CHRONIC DISEASE \| 9 \| \| 408_10 \| REFUSED/NO ANSWER \| 10 \|   408_9a: IF OTHER SPECIFY: _______________________ | | |  | |
| 409 | | Have you ever been diagnosed with above mentioned that might be diabetes complications?  **(MULTIPLE ANSWERS ALLOWED)** | | | \| 409_1 \| HYPERTENSION \| 1 \| \| --- \| --- \| --- \| \| 409_2 \| CARDIO-VASCULAR DISEASE \| 2 \| \| 409_3 \| STROKE \| 3 \| \| 409_4 \| EYE DAMAGE \| 4 \| \| 409_5 \| FOOT DAMAGE \| 5 \| \| 409_6 \| NERVE DAMAGE \| 6 \| \| 409_7 \| KIDNEY DAMAGE \| 7 \| \| 409_8 \| SKIN CONDITIONS \| 8 \| \| 409_9 \| HEARING PROBLEMS \| 9 \| \| 409_10 \| DEMENTIA \| 10 \| \| 409_11 \| DEPRESSION \| 11 \| \| 409_12 \| SEXUAL PROBLEMS \| 12 \| \| 409_13 \| OTHER \| 13 \| \| 409_14 \| REFUSED/NO ANSWER \| 14 \| \| 409_13a: IF OTHER, SPECIFY: ___________________ \| \| \| \| | | |  | |
| 410 | | What type of medication do you take for your diabetes?  **(ONLY ONE ANSWER ALLOWED)** | | | \| NO MEDICATION \| 1 \| \| --- \| --- \| \| ORAL MEDICATION \| 2 \| \| INSULIN INJECTIONS \| 3 \| \| BOTH \| 4 \| \| REFUSED \| 5 \| | | | IF 1 ⇒ **SECTION 5** | |
| 410a | | Do you take medicine prescribed by doctor or buy yourself?  **(MULTIPLE OPTIONS ALLOWED)** | | | \| 410a_1 \| TAKE MEDICINE PRESCRIBED BY DOCTOR \| 1 \| \| --- \| --- \| --- \| \| 410a_2 \| BUY MEDICINE MYSELF \| 2 \| \| 410a_3 \| REFUSED/ NO ANSWER \| 3 \| | | | IF 410a_2  **⇒ 410b/c**  IF 410a_1 or 3  **⇒ 411** | |
| 410b | | IF you buy medicine yourself, what kinds of medicines do you take?  **(MULTIPLE ANSWERS ALLOWED)** | | | \| 410b_1 \| VIETNAMESE HERBAL MEDICINE \| 1 \| \| --- \| --- \| --- \| \| 410b_2 \| CHINESE HERBAL MEDICINE \| 2 \| \| 410b_3 \| INSULIN \| 3 \| \| 410b_4 \| ORAL MEDICATION \| 4 \| \| 410b_5 \| FUNCTIONAL FOODS \| 5 \| \| 410b_6 \| OTHER \| 6 \|   410b_6a: IF OTHER, SPECIFY: ___________________ | | |  | |
| 410c | | Why did do you buy diabetes medicine besides that prescribed by the doctor?  **(MULTIPLE ANSWERS ALLOWED)** | | | \| 410c_1 \| DON’T TRUST THE QUALITY OF INSURANCE MEDICINE \| 1 \| \| --- \| --- \| --- \| \| 410c_2 \| THINK THE INSURANCE MEDICATION IS LESS EFFECTIVE \| 2 \| \| 410c_3 \| PICK UP THE INSURANCE MEDICINE TAKE TOO MUCH TIME \| 3 \| \| 410c_4 \| INSURANCE MEDICINE HAS MORE SIDE EFFECTS \| 4 \| \| 410c_5 \| OUT OF MEDICINE, NO TIME TO TAKE FROM HOSPITAL \| 5 \| \| 410c_6 \| OUT OF MEDICINE, NO MONEY TO BUY OR NO TIME TO BUY \| 6 \| \| 410c_7 \| HERBS HAVE FEWER SIDE EFFECTS \| 7 \| \| 410c_8 \| OTHERS ENCOURAGE ME TO TAKE IT \| 8 \| \| 410c_9 \| GIVEN BY OTHER PEOPLE \| 9 \| \| 410c_10 \| OTHER \| 10 \| \| 410c_11 \| REFUSED/NO ANSWER \| 11 \| \| 410c_10a IF OTHER, SPECIFY:_______________________ \| \| \| \| | | |  | |
| 411 | | Have you ever changed the doses of diabetes medicine?  **(MULTIPLE ANSWERS ALLOWED)**  ***(Note if patients forget to take medicine)*** | | | \| 411_1 \| NEVER \| 1 \| \| --- \| --- \| --- \| \| 411_2 \| INCREASING DOSE \| 2 \| \| 411_3 \| REDUCING DOSE \| 3 \| \| 411_4 \| DON’T REMEMBER \| 4 \| | | |  | |
| 412 | | What would you like to know more about in relation to your diabetes? | | | \|  \|  \| NO \| YES \| \| --- \| --- \| --- \| --- \| \| 412_1 \| MEDICATION \| 0 \| 1 \| \| 412_2 \| DIET \| 0 \| 1 \| \| 412_3 \| EXERCISE \| 0 \| 1 \| \| 412_4 \| FOOT CARE \| 0 \| 1 \| \| 412_5 \| COMPLICATIONS \| 0 \| 1 \| \| 412_6 \| SYMPTOMS OF HYPOGLYCEMIA \| 0 \| 1 \| \| 412_7 \| SYMPTOMS OF HYPERGLYCEMIA \| 0 \| 1 \| \| 412_8 \| HOW TO STAY EMOTIONALLY BALANCED \| 0 \| 1 \| \| 412_9 \| HOW TO INTEGRATE DIABETES INTO THE FAMILY \| 0 \| 1 \| \| 412_10 \| Other \| 0 \| 1 \|   If other, please specify: ________________________ | | |  | |
| **SECTION 7**  **SELF-MANAGEMENT** | | | | | | | | | |
| The following questions regarding how you manage diabetes yourself | | | | | | | | | |
| 701 | | | How often do you check your blood pressure value in the last 4 weeks? | | \| NEVER \| 1 \| \| --- \| --- \| \| RARELY \| 2 \| \| DAILY OR NEARLY EVERY DAY \| 3 \| \| ONE TO THREE TIMES A MONTH \| 4 \| \| ONCE OR TWICE A WEEK \| 5 \| \| DON’T REMEMBER \| 6 \| \| REFUSED/NO ANSWER \| 7 \| | | | **IF NEVER 🡺 702** | |
| 701a | | | **Where** do you often do this?  **(ONLY ONE ANSWER ALLOWED)** | | \| IN MY HOUSE \| 1 \| \| --- \| --- \| \| NEIGHBOR’S HOUSE \| 2 \| \| FRIEND’S HOUSE \| 3 \| \| HOUSE OF RELATIVES \| 4 \| \| COMMUNE HEALTH STATION \| 5 \| \| OTHER \| 6 \|   701a_6a: IF OTHER, SPECIFY: ________________________ | | |  | |
| 702 | | | Do you know how to take care of your fof oot? | | \| NO \| YES \| REFUSED/NO ANSWER \| \| --- \| --- \| --- \| \| 0 \| 1 \| 9 \| | | | **IF NEVER 🡺 703** | |
| 702a | | | How do you care your foot?  **(MULTIPLE ANSWERS ALLOWED)** | | \| 703a_1 \| WASHING FOOT EVERYDAY \| 1 \| \| --- \| --- \| --- \| \| 703a_2 \| USING CREAM \| 2 \| \| 703a_3 \| MASSAGES \| 3 \| \| 703a_4 \| ACUPUNCTURE \| 4 \| \| 703a_5 \| DON’T BAREFOOT \| 5 \| \| 703a_6 \| SOAKING FOOT WITH HERBS \| 6 \| \| 703a_7 \| OTHER \| 7 \| \| 703a_8 \| DON’T REMEMBER \| 8 \| \| 703a_9 \| REFUSED/ NO ANSWER \| 9 \| \| 703a_7a: IF OTHER, SPECIFY: ___________________ \| \| \| \| | | |  | |
| 703 | | | Can you feel if you had **HYPOGLYCEMIA**? | | \| NO \| YES \| REFUSED/NO ANSWER \| \| --- \| --- \| --- \| \| 0 \| 1 \| 9 \| | | | **IF NO 🡺 704** | |
| 703a | | | If yes, In your perception, how many times **in the last four weeks** have you had a **HYPOGLYCEMIA reaction** with symptoms such as sweating, confusion, headache, fatigue, dizziness, easily angry? | | \| NO \| 1 \| \| --- \| --- \| \| 1-3 TIMES \| 2 \| \| 4 TIMES OR MORE \| 3 \| \| DON’T REMEMBER \| 4 \| \| REFUSED/ NO ANSWER \| 5 \| | | |  | |
| 703b | | | How did you deal with **HYPOGLYCEMIA**?  **(MULTIPLE ANSWERS ALLOWED)** | | \| 703b_1 \| DO NOTHING \| 1 \| \| --- \| --- \| --- \| \| 703b_2 \| GO TO COMMUNE HEALTH STATION \| 2 \| \| 703b_3 \| CHECK **BLOOD GLUCOSE** \| 3 \| \| 703b_4 \| DRINK PURE WATER \| 4 \| \| 703b_5 \| DRINK 1 CUP OF FRUIT JUICE \| 5 \| \| 703b_6 \| DRINK MILK \| 6 \| \| 703b_7 \| EAT 1 – 2 PIECES OF CANDY \| 7 \| \| 703b_8 \| EAT FRUIT WITH LOW SUGAR \| 8 \| \| 703b_9 \| OTHER \| 9 \| \| 703b_10 \| DON’T REMEMBER \| 10 \| \| 703b_11 \| REFUSED/ NO ANSWER \| 11 \| \| 703b_9a: IF OTHER, SPECIFY: ___________________ \| \| \| \| | | |  | |
| 704 | | | Can you feel if you had **HYPERGLYCEMIA**? | | \| NO \| YES \| REFUSED/ NO ANSWER \| \| --- \| --- \| --- \| \| 0 \| 1 \| 9 \| | | | **IF NO**  ⇒ 705 | |
| 704a | | | If yes, In your perception, how many times **in the last four weeks** have you had a **HYPERGLYCEMIA reaction** with symptoms such as increased thirst, frequent peeing, headache, and fatigue. | | \| NO \| 1 \| \| --- \| --- \| \| 1-3 TIMES \| 2 \| \| 4 TIMES OR MORE \| 3 \| \| DON’T REMEMBER \| 4 \| \| REFUSED/ NO ANSWER \| 5 \| | | |  | |
| 704b | | | How did you deal with **HYPERGLYCEMIA**?  **(MULTIPLE ANSWERS ALLOWED)** | | \| 704b_1 \| DO NOTHING \| 1 \| \| --- \| --- \| --- \| \| 704b_2 \| GO TO COMMUNE HEALTH STATION \| 2 \| \| 704b_3 \| CHECK BLOOD GLUCOSE \| 3 \| \| 704b_4 \| INJECT INSULIN IMMEDIATELY \| 4 \| \| 704b_5 \| TAKE MEDICATION \| 5 \| \| 704b_6 \| DRINK MORE WATER \| 6 \| \| 704b_7 \| OTHER \| 7 \| \| 704b_8 \| DON’T REMEMBER \| 8 \| \| 704b_9 \| REFUSED/ NO ANSWER \| 9 \| \| 704b_7a: IF OTHER, SPECIFY: ___________________ \| \| \| \| | | |  | |
| 705 | | | Do you have access to a glucometer outside a health facility? | | \| NO \| YES \| REFUSED/NO ANSWER \| \| --- \| --- \| --- \| \| 0 \| 1 \| 9 \| | | | **IF NO**  ⇒ 705e | |
| 705a | | | **IF YES**: where?  **(MULTIPLE ANSWERS ALLOWED)** | | \| 705a_1 \| IN MY HOUSE \| 1 \| \| --- \| --- \| --- \| \| 705a_2 \| NEIGHBOR \| 2 \| \| 705a_3 \| FRIEND \| 3 \| \| 705a_4 \| HOUSE OF RELATIVES \| 4 \| \| 705a_5 \| OTHER \| 5 \|   705a_5a: IF OTHER, SPECIFY: _______________________ | | |  | |
| 705b | | | How often do you measure your blood sugar per month outside a health facility?  **(ONLY ONE ANSWER ALLOWED)** | | \| RARELY \| 1 \| \| --- \| --- \| \| DAILY OR NEARLY EVERY DAY \| 2 \| \| ONCE OR TWICE A WEEK \| 3 \| \| ONE TO THREE TIMES A MONTH \| 4 \| \| DON’T REMEMBER \| 5 \| \| REFUSED/NO ANSWER \| 6 \| | | |  | |
| 705c | | Who **usually** measures your blood sugar?  **(MULTIPLE ANSWERS ALLOWED)** | | | \| 705c_1 \| I DO IT MYSELF \| 1 \| \| --- \| --- \| --- \| \| 705c_2 \| MY SUPPORTER \| 2 \| \| 705c_3 \| FRIEND \| 3 \| \| 705c_4 \| NEIGHBOR \| 4 \| \| 705c_5 \| OTHER DIABETICS \| 5 \| \| 705c_6 \| OTHER \| 6 \| \| 705c_7 \| REFUSED/NO ANSWER \| 7 \|   705c_6a: IF OTHER, SPECIFY: __________________________ | | |  | |
| 705d | | The value at last checkup | | | _____________________(mmol/L) (DONT REMEMBER: 99) | | |  | |
| 705e | | Would you like to own a glucometer at home/share a glucometer with other diabetics? | | | \| NO \| YES \| REFUSED/NO ANSWER \| \| --- \| --- \| --- \| \| 0 \| 1 \| 9 \| | | | **IF 705a=1 🡺 706**  **IF NO** ⇒ **706** | |
| 705f | | Who **SHOULD** test your blood sugar?  **(ONLY ONE ANSWER ALLOWED)** | | | \| I DO IT MYSELF \| 1 \| \| --- \| --- \| \| MY SUPPORTER \| 2 \| \| FRIEND \| 3 \| \| NEIGHBOR \| 4 \| \| OTHER DIABETICS \| 5 \| \| OTHER \| 6 \| \| REFUSED/NO ANSWER \| 7 \|   705f_1a: IF OTHER, SPECIFY: __________________________ | | |  | |
| 706 | | Have you ever smoked? | | | \| NEVER SMOKED \| SMOKED PREVIOUSLY \| PRESENT SMOKER \| \| --- \| --- \| --- \| \| 1 \| 2 \| 3 \| | | | IF 3 🡺 706a | |
| 706a | | How many cigarettes do you smoke per day? | | | \| <5 CIGARETTES \| 1 \| \| --- \| --- \| \| 5-10 CIGARETTES \| 2 \| \| 11 - 20 CIGARETTES \| 3 \| \| 21 - 30 CIGARETTES \| 4 \| \| >31 CIGARETTES \| 5 \| \| DON’T REMEMBER \| 6 \| \| REFUSED/NO ANSWER \| 7 \| | | |  | |
| 707 | | How often did you drink alcohol last month?  (Read up options) | | | \| NEVER \| 1 \| \| --- \| --- \| \| RARELY \| 2 \| \| DAILY OR NEARLY EVERY DAY \| 3 \| \| ONCE OR TWICE A WEEK \| 4 \| \| ONE TO THREE TIMES A MONTH \| 5 \| \| DON’T REMEMBER \| 6 \| \| REFUSED/NO ANSWER \| 7 \| \| OTHER \| 8 \|   707_1a: IF OTHER, SPECIFY:____________________ | | | IF NEVER 🡺 **SECTION 8** | |
| 707a | | In a typical week, how much beer/wine do you drink?  (1 standard cup = 330 ml beer 4% = 120 ml wine 11.5% = 30 ml spirit 40%) | | | \| < 1 CUP \| 1 \| \| --- \| --- \| \| 1 – 2 CUPS \| 2 \| \| 3 – 5 CUPS \| 3 \| \| 6 – 10 CUPS \| 4 \| \| >10 CUPS \| 5 \| \| DON’T REMEMBER \| 6 \| \| REFUSED/NO ANSWER \| 7 \| | | |  | |
